# Long‐Range Polariton Canalization in a Graphene‐Nanoribbon–Covered MgTeMoO_6_ Metasurface

**DOI:** 10.1002/nap2.70113

**Published:** 2026-04-29

**Authors:** Chia‐Chien Huang

**Affiliations:** ^1^ Department of Physics National Chung Hsing University Taichung Taiwan; ^2^ Graduate Institute of Nanoscience National Chung Hsing University Taichung Taiwan

**Keywords:** hyperbolic dispersion, long‐range, metasurface, polariton canalization, tellurite quaternary oxide, topological transition, van der Waals

## Abstract

van der Waals (vdW) quaternary oxide structures, such as MgTeMoO_6_ slabs and nanoribbons, have recently emerged as promising platforms for demonstrating polariton propagation with hyperbolic dispersion and canalization. This expands the catalog of exceptional optical and electronic vdW materials. By leveraging topological transitions, various vdW heterostructures can induce extraordinary in‐plane canalized polaritons. Yet, achieving long‐range canalized polariton propagation has remained a significant challenge. In this study, we introduce a novel approach by covering the ridge tops of an MgTeMoO_6_ metasurface with graphene ribbons. This configuration significantly extends the propagation length of canalized polaritons to an unprecedented 7.95 μm, which is more than 20 times the propagation distance observed in a bare MgTeMoO_6_ metasurface. This enhancement is achieved through the coherent coupling of graphene plasmon polaritons and MgTeMoO_6_ phonon polaritons, forming a guided phase‐locked mode that minimizes energy losses. Notably, the resulting canalized polaritons exhibit exceptionally long lifetimes of around 86 picoseconds, surpassing previously reported vdW platforms. Simulations using an antenna‐launched setup confirm the experimental feasibility of our design. These findings open new avenues for applying polaritonic vdW platforms in nanophotonics, super‐resolution imaging, and diffraction‐less hyperlensing in the mid‐infrared regime.

## Graphical abstract

Simulated Re(*E*_*z*_) field distributions at *ω* = 924 cm^−1^ for the graphene grating, MgTeMoO_6_ grating, the proposed hybrid structure, and the corresponding IFCs in (*k*_*x*_, *k*_*y*_) space are shown. Re(*E*_*z*_) field maps in the *x*–*y* plane at *ω* = 924 cm^−1^ for *E*_*f*_ = 50, 70, 74, 76, 80, and 88 meV.

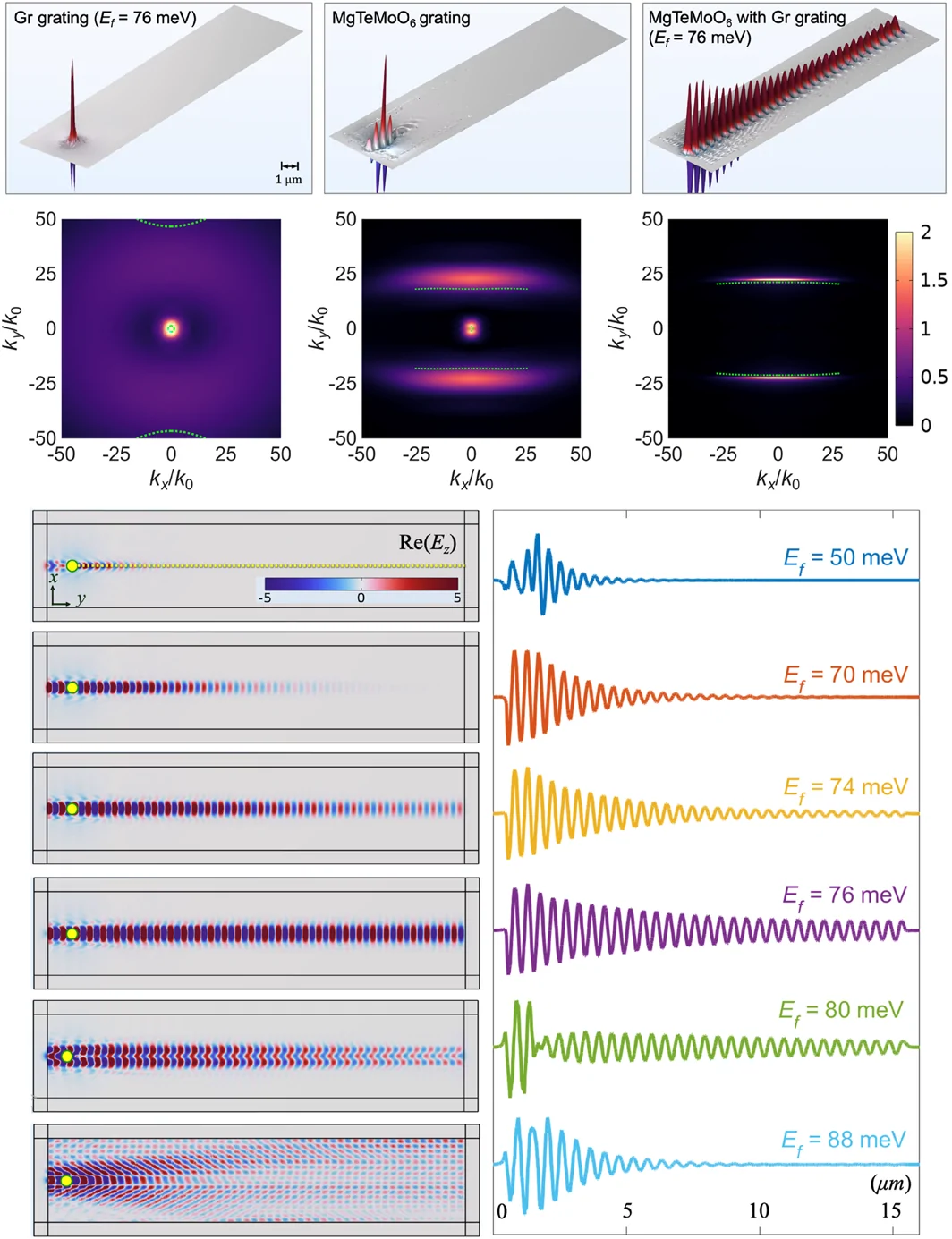

## 1 Introduction

Polariton canalization [1, 2, 3], namely the highly collimated propagation of electromagnetic energy, enables precise nanoscale light control without conventional waveguide geometries. It occurs when hybrid light–matter quasiparticles—formed by coupling photons with plasmons (plasmon polaritons, PPs) [4, 5, 6, 7, 8] or phonons (phonon polaritons, PhPs) [9, 10, 11, 12, 13]—undergo topological transitions [14, 15, 16, 17, 18] between hyperbolic and elliptical isofrequency contours (IFCs) in momentum space. Owing to its ability to guide energy in a diffraction‐suppressed manner, polariton canalization has attracted broad interest for applications in nanophotonics [19, 20, 21], super‐resolution imaging [22, 23], and thermal management [24, 25, 26]. In recent years, canalized polaritons have been demonstrated in a variety of van der Waals (vdW) materials and heterostructures, including twisted multilayer *α*‐MoO_3_ [27, 28, 29], graphene‐covered *α*‐MoO_3_ slabs [30, 31], and *α*‐MoO_3_ films on SiC substrates [23, 32]. Similar phenomena have also been reported in other vdW systems, such as twisted *α*‐V_2_O_5_ [33], LiV_2_O_5_ [34], and YVO_4_ flakes [35]. Some studies have explored single‐layer canalization [36, 37], canalization enabled by hybridization [38], and related applications in energy management [39]. In parallel, metasurfaces based on hexagonal boron nitride (hBN) [40, 41, 42] have provided a flexible platform for engineering IFCs and tailoring wave dynamics.

Beyond conventional vdW materials, Sun et al. [43] recently introduced a family of tellurite molybdenum quaternary oxides, ATeMoO_6_ (A = Mg, Zn, Mn, Cd, Co, etc.), featuring strong nonlinear optical, piezoelectric, and electro‐optical responses. Motivated by the hBN metasurface concept [41, 42], we previously investigated MgTeMoO_6_ metasurfaces and demonstrated canalized polaritons in the 921–928 cm^−1^ spectral range [44], thus broadening the platform landscape for vdW canalization. However, despite their deep‐subwavelength confinement, these canalized modes propagate over only a few polariton wavelengths, which hinders their use in practical photonic integrated circuits.

Here, we propose a hybrid structure in which graphene nanoribbons are placed on the ridge tops of an MgTeMoO_6_ metasurface. This configuration enhances the STO resonance of canalized polaritons propagating across the MgTeMoO_6_ ribbons. Through coupling between surface plasmon polaritons (SPPs) in the graphene nanoribbons and phonon polaritons (PhPs) in the MgTeMoO_6_ metasurface, hybrid plasmon–phonon polaritons (HPPhPs) [45, 46, 47] with greatly extended propagation can be achieved by properly tuning the graphene Fermi level. Unlike previous designs, we find that modest values are sufficient to realize long‐range canalized propagation. We further analyze the key canalization characteristics to quantify the performance enhancement provided by the hybrid structure. The effects of geometric parameters and graphene Fermi level are systematically investigated and optimized, and the fabrication feasibility is also discussed.

## 2 Physical Mechanism for Long‐Range Canalized Polaritons

High‐quality vdW MgTeMoO_6_ crystals have been experimentally grown on Si/SiO_2_ substrates using a self‐serve melt method [43, 48]. In this platform, MgTeMoO_6_ exhibits hyperbolic dispersion in the spectral range *ω* = 895–903 cm^−1^, characterized by opposite‐sign in‐plane permittivities along the *x*‐[100] (*ε*_*x*_ < 0) and *y*‐[010] (*ε*_*y*_ > 0) directions. The frequency‐dependent permittivity tensor *ε*(*ω*) is modeled using a Lorentz oscillator form [43].

(1) $$\varepsilon_{i}\left(\omega \right)=\varepsilon_{\infty ,i}+\sum_{j=1}^{3}\left(\frac{f_{ij}}{\omega_{ij}^{2}-\omega^{2}-i\omega \gamma_{ij}}\right),\left(i=x,y,z\right)\;$$

where *ε*_∞,*i*_ represents the high‐frequency permittivity along the *i* direction, whereas *f*_*ij*_, *ω*_*ij*_, and *γ*_*ij*_ are the oscillator strength, resonance frequency, and damping rate of the *j*‐th Lorentz term along *i*, respectively. The constants used in this study are *ε*_∞,*x*_ = 3.0, *ε*_∞,*y*_ = 3.1, and *ε*_∞,*z*_ = 0.7; *f*_*x*1_ = 570.04, *f*_*x*2_ = 90.3, *f*_*x*3_ = 420.63, *f*_*y*1_ = 710.71, *f*_*y*2_ = 140.53, *f*_*y*3_ = 316.5, and *f*_*z*1_ = 103.37; *ω*_*x*1_ = 698.47 cm^−1^, *ω*_*x*2_ = 749.47 cm^−1^, *ω*_*x*3_ = 895.5 cm^−1^, *ω*_*y*1_ = 684.54 cm^−1^, *ω*_*y*2_ = 749.08 cm^−1^, *ω*_*y*3_ = 902.98 cm^−1^, and *ω*_*z*1_ = 950 cm^−1^; *γ*_*x*1_ = 6.09, *γ*_*x*2_ = 36.71, *γ*_*x*3_ = 1.81, *γ*_*y*1_ = 2.81, *γ*_*y*2_ = 31.8, *γ*_*y*3_ = 1.99, and *γ*_*z*1_ = 7. Hyperbolic propagating PhPs in MgTeMoO_6_ flakes have been directly observed via infrared nanoimaging and microscopy. We previously demonstrated that an MgTeMoO_6_ metasurface on a gold (Au) substrate can support canalized polaritons in the range *ω* = 921–928 cm^−1^ [44], inspired by hBN metasurfaces that exhibit emergent canalized polariton modes [41, 42]. However, practical implementations of polariton canalization across various platforms [27, 28, 29, 30, 31, 32, 33, 34, 41, 42, 43, 44] remain limited by short propagation lengths—typically only 2–5 polariton wavelengths. In our previous work [44], the MgTeMoO_6_ metasurface exhibited a propagation length of *L*_*p*_ = 0.3–0.40 μm (≈ 2*λ*_*p*_). To overcome this limitation, we propose a heterostructure in which graphene nanoribbons are placed on the ridge tops of the MgTeMoO_6_ metasurface. The structure is defined by the grating period (*P*), ribbon width (*w*), gap (*g*), and height (*h*), with duty cycle *η* = *w*/*P*, as shown in Figure 1a. We first describe the graphene nanoribbons. To incorporate nonlocal effects in electrically thin films, the air gaps between neighboring graphene ribbons are modeled using an effective surface conductivity *σ*_*c*_ = –2*iωε*_0_ (*P*/*π*) ln [csc(*πg*/2*P*)] [2, 41, 49, 50, 51], as commonly adopted in refs. [2, 37, 45, 46, 47]. The corresponding bulk effective permittivity *ε*_*c*_ is obtained through *ε*_*c*_ = *iσ*_*c*_/(*ωε*_0_ *h*). Using the modified effective medium approximation [36, 37, 38, 40, 52], the equivalent permittivity components of the graphene metasurface are obtained (Equations ((2), (3), (4))) as follows:

(2) $$\varepsilon_{eq,x}=\eta \varepsilon_{x}+\left(1-\eta \right)\varepsilon_{a},$$

(3) $$\varepsilon_{eq,y}={\left[\frac{\eta }{\varepsilon_{y}}+\frac{\left(1-\eta \right)}{\varepsilon_{c}}\right]}^{-1},$$

(4) $$\varepsilon_{eq,z}=\eta \varepsilon_{z}+\left(1-\eta \right)\varepsilon_{a}$$

where *ε*_*x*_, *ε*_*y*, and_ *ε*_*z*_ denote the permittivity components of monolayer graphene along the *x*, *y*, and *z* directions, respectively. For *p* = 75 nm, *w* = 45 nm, *g* = 30 nm, (*η* = 0.6), *h* = 100 nm, and carrier mobility *μ* = 0.5 m^2^/V·s, we plot the real parts of the effective permittivities *ε*_*eq,x*_ and *ε*_*eq,y*_ versus the graphene Fermi level *E*_*f*_ for *ω* = 922, 925, and 927 cm^−1^ (Figure 1b). The curves in the range *ω* = 922–927 cm^−1^ nearly overlap, indicating weak frequency dependence within the operating band. Notably, *ε*_*eq,y*_ is significantly larger than *ε*_*eq,x*_ over the range 70 meV < *E*_*f*_ < 110 meV, implying that canalized polariton propagation along the *y* direction in the bare MgTeMoO_6_ metasurface can be strongly enhanced by the graphene ribbons [2, 50].

**FIGURE 1:**
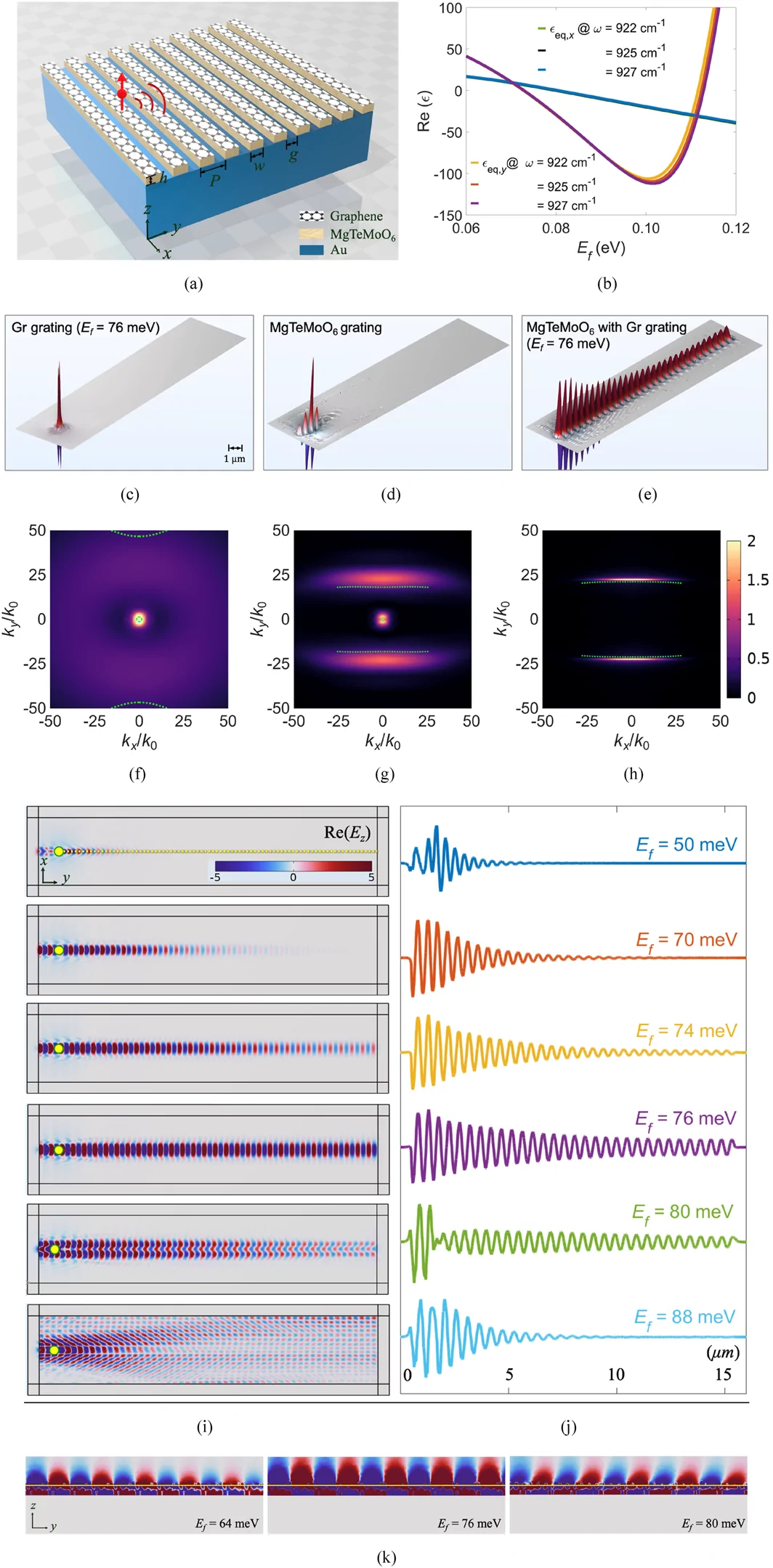
Structure design and canalized polaritons in the proposed platform. (a) Schematic of the MgTeMoO_6_ metasurface covered with graphene nanoribbons on an Au substrate. The double‐arrow symbol denotes the position and polarization of the *z*‐oriented dipole source. (b) Real parts of the effective permittivity components, *ε*_*eq,x*_ and *ε*_*eq,y*_, as a function of the graphene Fermi level (*E*_*f*_) for *ω* = 922, 925, and 927 cm^−1^. (c, d, e) Simulated Re(*E*_*z*_) field distributions at *ω* = 924 cm^−1^ for the graphene grating (c), MgTeMoO_6_ grating (d), the proposed hybrid structure (e). Their corresponding IFCs in (*k*_*x*_, *k*_*y*_) space are shown in (f), (g), and (h), respectively. (i) Re(*E*_*z*_) field maps in the *x*–*y* plane at *ω* = 924 cm^−1^ for *E*_*f*_ = 50, 70, 74, 76, 80, and 88 meV), where yellow markers indicate the dipole‐moment position and the yellow dotted line indicates the path of the 1D field profile in (j). (j) Corresponding 1D Re(*E*_*z*_) profiles along the *y* direction (right panels). (k) Re(*E*_*z*_) field profiles in the *y*–*z* plane for *E*_*f*_ = 64, 76, and 80 meV (left to right), illustrating the coupling evolution. All results are obtained with *p* = 75 nm, *w* = 45 nm, *g* = 30 nm, and *h* = 100 nm.

Before evaluating the performance of the proposed structure, we first discuss the fabrication process as follows: (1) High‐quality MgTeMoO_6_ crystals can be prepared via chemical flux growth, followed by mechanical exfoliation to obtain thin vdW flakes, as recently demonstrated for this material system [43]. The exfoliated flakes can then be deterministically transferred onto the Au substrate using established all‐dry viscoelastic stamping techniques, which provide high placement accuracy and avoid contamination [53]. (2) The MgTeMoO_6_ grating with sub‐100‐nm periodicity can be defined using high‐resolution electron‐beam lithography (EBL) followed by hard‐mask‐assisted dry etching or ion milling. Similar approaches have been successfully employed to fabricate nanostructured vdW metasurfaces with lateral feature sizes down to ∼100 nm [54, 55], indicating that the target period of ∼75 nm is within current nanofabrication capability. (3) To address the key challenge of graphene nanoribbon alignment, a widely used approach is to first transfer a continuous monolayer graphene sheet onto the pre‐patterned MgTeMoO_6_ grating using deterministic dry transfer [56]. Subsequently, the graphene nanoribbon array can be defined using a second aligned EBL step widths down to tens of nanometers [57, 58] followed by oxygen plasma etching. In this approach, the alignment is handled lithographically via alignment markers, rather than mechanically, significantly improving feasibility and accuracy. (4) The metallic launch antenna can be fabricated in a subsequent aligned EBL step followed by Ti/Au evaporation and lift‐off, consistent with established antenna‐launched polariton experiments [59, 60]. We emphasize here that the proposed structure can be realized by combining well‐established process modules that have already been demonstrated individually in van der Waals (vdW) polaritonic and graphene nanofabrication platforms.

All numerical simulations were performed using COMSOL Multiphysics with perfectly matched layers surrounding the computational domain. A *z*‐polarized electric dipole located 100 nm above the graphene layer was used to excite polaritons. The calculated Re(*E*_*z*_) fields at 50 nm above the top surface are shown for the bare graphene grating, bare MgTeMoO_6_ grating, and the proposed hybrid structure in Figure 1c–e, respectively, and the corresponding IFCs in (*k*_*x*_, *k*_*y*_) spaces with the analytical solutions indicated by the green dotted lines are shown in Figure 1f–h, for *p* = 75 nm, *w* = 45 nm, *g* = 30 nm, *h* = 100 nm, *ω* = 924 cm^−1^, and *E*_*f*_ = 76 meV. First, a localized graphene resonance at low *E*_*f*_ is strongly confined near the excitation region (see Figure 1c). We observe that the IFC contains broad momentum content, including large‐*k* components and behaving as a localized plasmonic resonator simultaneously. Secondly, the IFC of the MgTeMoO_6_ grating contains three main features (see Figure 1g): a strong central bright spot near (*k*_*x*_/*k*_0_, *k*_*y*_/*k*_0_) ≈ (0, 0), plus two broader horizontal branches around *k*_*y*_/*k*_0_ ≈± 19. The central spot indicates a low‐momentum and localized *E*_*z*_ field concentrated near the excitation region rather than fully converted into directional high‐*k* propagation. The upper and lower branches are broader and more diffuse, so the allowed momenta are less sharply selected. This implies stronger mode scattering, larger momentum spread, and therefore weaker directional transport. When integrating graphene grating with the MgTeMoO_6_ one, this localized graphene response efficiently enhances the MgTeMoO_6_‐supported canalized PhP. As a result, the hybrid structure redistributes the spectral weight from the source‐centered low‐*k* component into the finite‐*k* canalized channel, resulting in the suppressed central feature of IFC (see Figure 1h). We observe that the spectrum is dominated by two very thin, bright horizontal branches at *k*_*y*_/*k*_0_ ≈± 22. The much narrower linewidth in *k*‐space indicates a more selective polaritonic mode, typically associated with better directional propagation, stronger canalization, and less laterally spreading beam.

To further elucidate the mechanism, we investigate how *E*_*f*_ controls the propagation characteristics. Figure 1i presents 2D Re(*E*_*z*_) maps in the x–*y* plane for *E*_*f*_ = 50, 70, 74, 76, 80, and 88 meV (top to bottom), and the corresponding 1D profiles along *y* are shown in Figure 1j. As *E*_*f*_ increases, the beam width increases moderately, consistent with weaker mode confinement. For *E*_*f*_ = 20–50 meV, the Re(*E*_*z*_) profiles remain nearly unchanged and indicate strong interband absorption due to insufficient Pauli blocking (ℏ*ω* < 2*E*_*f*_). When *E*_*f*_ is increased to 76 meV, the *L*_*p*_ reaches an optimum value of *L*_*p*_ = 7.94 μm. At *E*_*f*_ = 80 meV, the fundamental mode transitions to a higher‐order mode, and the field becomes more spatially distributed across the surface for *E*_*f*_ = 88 meV. This *E*_*f*_ dependence can be understood from the coupling condition between SPPs and PhPs, as shown by the *y*–*z* plane field profiles in Figure 1k for *E*_*f*_ = 64, 76, and 80 meV (yellow lines denote graphene positions). At *E*_*f*_ = 76 meV, the Re(*E*_*z*_) pattern above the graphene repeats with nearly uniform amplitude along *y* (middle panel of Figure 1k), indicating coherent coupling [10] enabled by dispersion tuning of the graphene SPPs. In this regime, radiation leakage into the far field is suppressed and losses are dominated primarily by material absorption. By contrast, the field amplitude decays rapidly, and the above‐graphene interference pattern is tilted with respect to the *z* axis at *E*_*f*_ = 64 and 80 meV, revealing a phase mismatch between fields above and below the graphene.

## 3 Spectral Response and Propagation Properties of Canalized Polaritons

Considering the spectral response, we plot the 1D Re(*E*_*z*_) profiles and their amplitude decay for the bare MgTeMoO_6_ metasurface (Figure 2a,b) and the proposed structure (Figure 2c,d) over *ω* = 922–927 cm^−1^, using *p* = 75 nm, *w* = 45 nm, *g* = 30 nm, and *h* = 100 nm. For the bare MgTeMoO_6_ metasurface, the propagation length is limited to *L*_*p*_ = 0.42–0.26 μm, corresponding to only ∼ 2–3*λ*_*p*_. In contrast, the proposed structure achieves *L*_*p*_ = 7.95 μm, enabling propagation beyond 20*λ*_*p*_. Since the 1D field profile along the propagation direction shows a damped oscillatory behavior, we extracted the decay rate from the envelope of the oscillation. In practice, the successive local maxima of the field profile (not the intensity) were selected to define the envelope, and these peak amplitudes were fitted with an exponential function, *E*_*z*_(*y*) ∝ exp(‐*αy*) [23, 29, 35]. From the fitted decay constant *α*, the propagation length was determined as *L*_*p*_ = 1/*α* = 1/Im(*k*_*p*_). To satisfy the coherent coupling, the optimal *E*_*f*_ must be adjusted with frequency: *E*_*f*_ = 87 meV (*ω* = 922 cm^−1^), 82 meV (923 cm^−1^), 76 meV (924 cm^−1^), 71 meV (925 cm^−1^), 67 meV (926 cm^−1^), and 63 meV (927 cm^−1^). As *ω* increases, a lower *E*_*f*_ is required to maintain coherent coupling. At *ω* = 927 cm^−1^, *L*_*p*_ decreases to 2.89 μm, which we attribute to increased interband absorption in graphene. We also present the 2D Re(*E*_*z*_) maps for both structures at *ω* = 923 and 925 cm^−1^. These results show that the proposed structure supports not only unprecedented long‐range propagation distance but also high‐quality canalized polaritons, albeit with a moderately larger beam width (∼380 nm) than that of the bare MgTeMoO_6_ metasurface (∼200 nm) [44].

**FIGURE 2:**
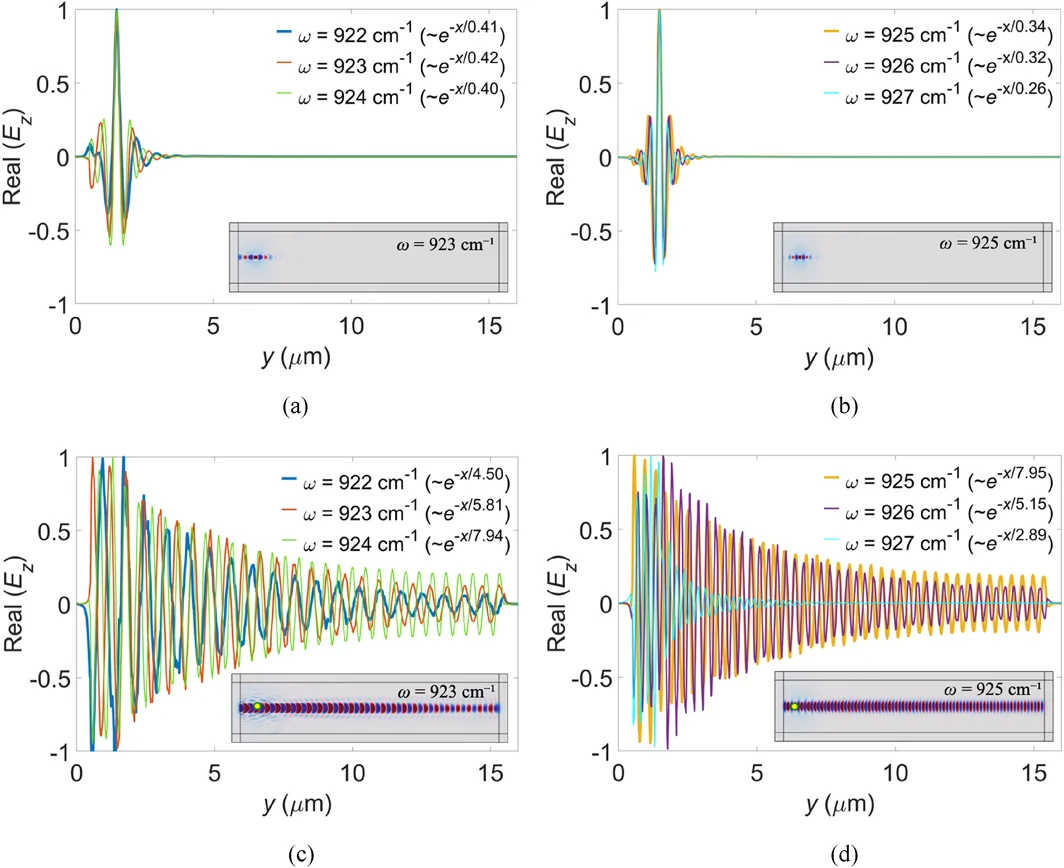
Spectral response of canalized polaritons. (a, b) 1D Re(*Ez*) profiles of the bare MgTeMoO6 metasurface at (a) *ω* = 922, 923, and 924 cm^−1^ and (b) *ω* = 925, 926, and 927 cm^−1^. (c, d) Corresponding 1D Re(*Ez*) profiles of the proposed structure (MgTeMoO6 metasurface with graphene nanoribbons) at (c) *ω* = 922 (*Ef* = 87 meV), 923 (*Ef* = 82 meV), and 924 cm^−1^ (*Ef* = 76 meV), and (d) *ω* = 925 (*Ef* = 71 meV), 926 (*Ef* = 67 meV), and 927 cm^−1^ (*Ef* = 63 meV), where *Ef* is tuned to satisfy the coherent coupling condition at each frequency. Insets show the 2D Re(*Ez*) field maps at *ω* = 923 and 925 cm^−1^.

To further characterize the canalized polaritons, we evaluate the mode confinement (defined as Re(*k*_*p*_)/*k*_0_, where *k*_0_ is the free‐space wavevector), propagation length *L*_*p*_, group velocity (*V*_*g*_ = *dω*/*dk*), and lifetime (*τ* = *L*_*p*_/*V*_*g*_) of the bare graphene grating, bare MgTeMoO_6_ grating, and hybrid structure, as summarized in Figure 3a–d, respectively, over the canalization band *ω* = 922–928 cm^−1^. As shown in the avoided‐crossing diagram in Figure 3a, constructed on the basis of coupled‐mode theory [61], the wavevector *k*_*p*_ of the graphene grating mode decreases as *ω* increases and *E*_*f*_ decreases, as obtained from the three‐layer analytical model [2]. This trend can be understood from the competition between the graphene kinetic‐inductive conductivity, (*σ*_*g*_ ∼ i *E*_*f*_/*ω*), and the gap‐capacitive displacement‐current conductivity, *σ*_*C*_ ∼ i (2*ωε*_0_​*P*/*π*) ln[csc(*π g*/2*P*)]. When *ω* increases while *E*_*f*_ decreases, the graphene inductive response becomes weaker, whereas the capacitive coupling across the gaps becomes stronger. As a result, the system is driven from a more inductive regime toward a more capacitive one in the across‐ribbon direction. In this regime, the stronger gap capacitance supplies a larger fraction of the required reactive phase storage, making the mode no longer require as large a *k*_*p*_ to sustain propagation across the ribbons. We further note that the crossing point of the two uncoupled modes, namely the bare graphene grating mode and the bare MgTeMoO_6_ grating mode, occurs near *ω* = 927 cm^−1^ in Figure 4a, corresponding to the approximate phase‐matching condition. In an ideal lossless strong‐coupling system, this would produce two clearly resolved hybrid branches with anti‐crossing. In the present case, however, the graphene‐related branch is substantially more lossy, so one of the hybrid eigenmodes becomes too strongly damped or too weakly excited to be clearly identified. Consequently, instead of a symmetric anti‐crossing, only the dominant low‐loss hybrid branch is clearly observed, which is a common situation in dissipative hybrid systems. The orange curve in Figure 4a therefore corresponds to this lower‐loss hybrid mode. Accordingly, the strongest hybridization occurs in between of *ω* = 925–926 cm^−1^. This result demonstrates how coupling modifies the modal dispersion and identifies the strongest‐coupling condition obtained from the present numerical results. In terms of propagation, the bare MgTeMoO_6_ metasurface suffers from strong attenuation with *L*_*p*_ < 0.4 μm, whereas the proposed structure achieves more than a 20‐fold enhancement in *L*_*p*_ at *ω* = 924 and 925 cm^−1^. The proposed structure also supports slower polariton transport, with *V*_*g*_ < 3 × 10^−4^ *c* throughout the canalization window (*c* is the speed of light in vacuum), which is smaller than values reported for platforms such as hBN, twisted *α*‐MoO_3_ bilayers, and *α*‐V_2_O_5_. The reduced *V*_*g*_ relative to the bare MgTeMoO_6_ metasurface implies an increased photonic density of states, which is favorable for enhancing the Purcell effect. Note that the graphene grating shows a negative *V*_*g*_ in the spectral range. Owing to the substantially extended *L*_*p*_, the proposed structure exhibits an unusually long lifetime, with *τ* > 30 ps across *ω* = 922–927 cm^−1^ and reaching *τ* = 86 ps at *ω* = 925 cm^−1^, thereby substantially strengthening light–matter interactions.

**FIGURE 3:**
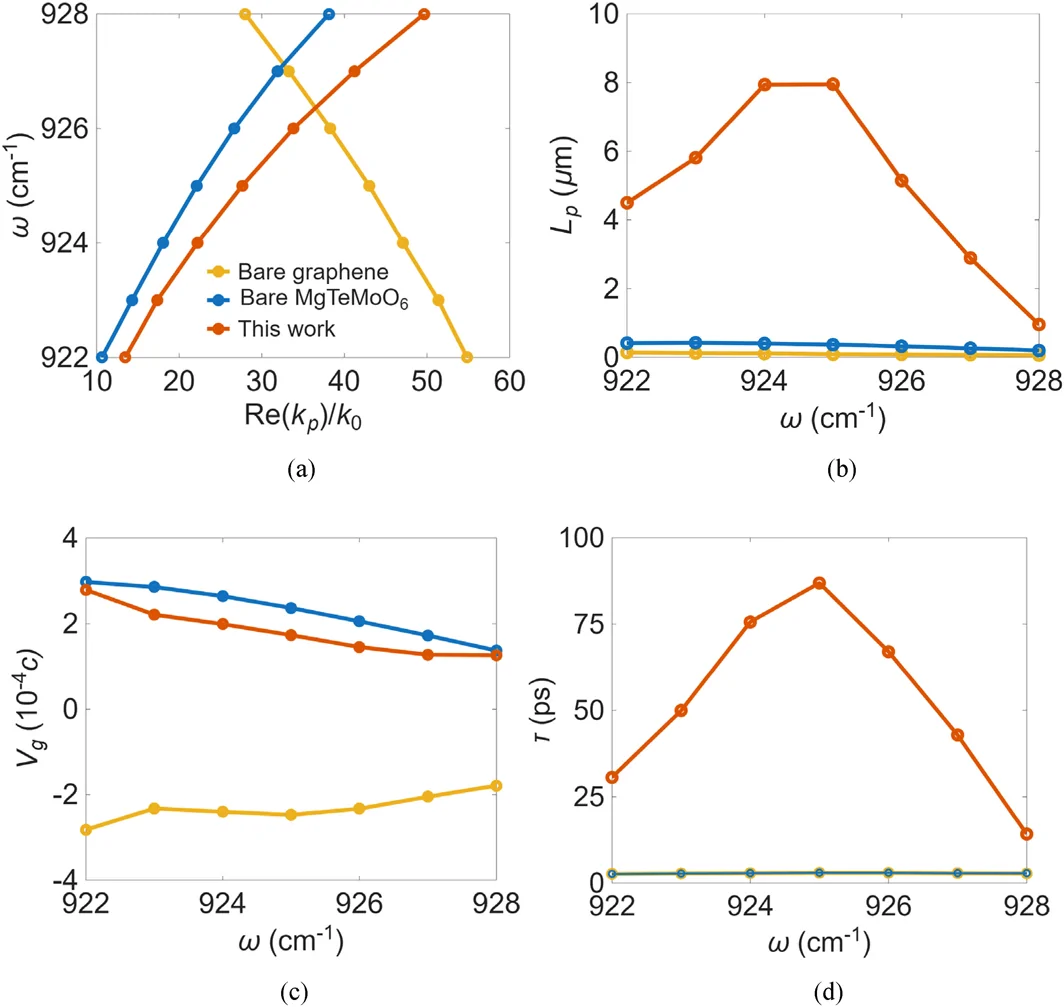
Frequency‐dependent canalization characteristics. Spectral dependence of key polaritonic metrics of the bare graphene grating, bare MgTeMoO_6_ grating, and hybrid structure: (a) mode confinement, (Re(*k*_*p*_)/*k*_0_); (b) propagation length, *L*_*p*_; (c) group velocity, *V*_*g*_; and (d) lifetime, *τ* at the geometric parameters: *p* = 75 nm, *w* = 45 nm, *g* = 30 nm, and *h* = 100 nm.

**FIGURE 4:**
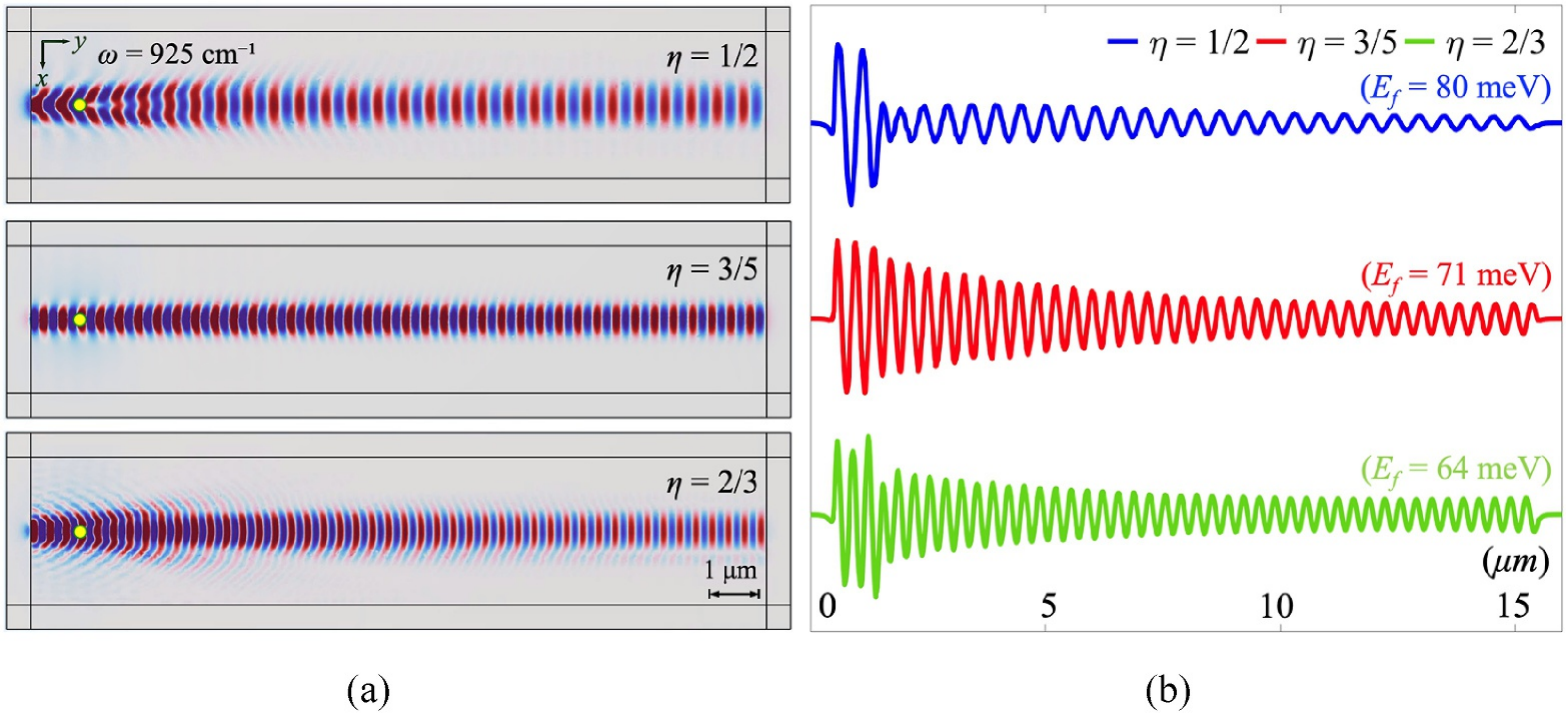
Duty‐cycle dependence of canalized polaritons. (a) Re(*Ez*) field maps in the *x*–*y* plane at *ω* = 925 cm^−1^ for duty cycles *η* = 1/2 (*Ef* = 80 meV), *η* = 3/5 (*Ef* = 71 meV), and *η* = 2/3 (*Ef* = 64 meV). (b) Corresponding 1D Re(*Ez*) profiles along the *y* direction, showing the propagation decay.

To evaluate performance, Table 1 compares the canalized‐polariton characteristics of the proposed structure (“This work”) with some vdW platforms reported previously [23, 27, 31, 34, 35, 41, 44], including the *L*_*p*_, *λ*_*p*_, *V*_*g*_, *τ*, and figure of merit (FoM = *L*_*p*_/*λ*_*p*_). Here, FoM quantifies the effective propagation distance in units of polariton wavelengths. With the exception of the YVO_4_ slab, most reported platforms exhibit *L*_*p*_ < 2 μm and *λ*_*p*_ < 0.4 μm, resulting in FoM < 5. Notably, although the YVO_4_ slab supports a long propagation length (*L*_*p*_ = 20.0 μm), its relatively large wavelength (*λ*_*p*_ = 4.70 μm) limits the FoM to below 5. By contrast, although the bare MgTeMoO_6_ metasurface exhibits a small FoM [40], introducing graphene nanoribbons on the ridge tops and tuning *E*_*f*_ enables a dramatic enhancement, yielding an unprecedented *L*_*p*_ = 7.95 μm and *τ* = 86 ps, and thus a record‐high FoM among the compared vdW canalization platforms.

**TABLE 1:** Comparison of propagation characteristics for canalized polaritons in various vdW platforms.

Structure	*α*‐MoO_3_/SiC [23]	T^a^ *α*‐MoO_3_ bilayers [27]	Gr^a^/*α*‐MoO_3_ [31]	LiV_2_O_5_ slab [34]	YVO_4_ slab [35]	hBN Grating [41]	MgTeMoO_6_ grating [44]	Graphene grating [62]	This work
*λ*_0_ (μm)	11.90	11.08	10.99	9.93	11.11	6.69	10.81	11.11	10.81
*L*_*p*_ (μm)	2.02	0.35	1.86	0.32	20.0	1.05	0.40	0.20	7.95
*λ*_*p*_ (μm)	0.42	0.28	0.44	0.37	4.70	0.31	0.41	1.40	0.38
*V*_*g*_ (10−^4^ *c*)	33.6	—	—	15	5.0	8.33	2.37	—	1.72
*τ* (ps)	2	—	—	0.73	13.3	4	8.8	—	86
FoM	∼ 5	1–2	4–5	∼ 1	4–5	3–4	∼ 1	∼ 0.2	∼ 20

^a^ T and Gr denote twisted and graphene‐covered configurations, respectively.

## 4 Geometry‐ and Material‐Dependent Canalization, Antenna‐Launched Polaritons, and Fabrication Tolerances

To investigate geometric effects, we plot the 2D Re(*E*_*z*_) distributions in the *x*–*y* plane for three duty cycles, *η* = 1/2, 3/5, and 2/3, under *p* = 75 nm, *h* = 100 nm, and *ω* = 925 cm^−1^ (Figure 4a). For each duty cycle, the canalization is optimized by selecting *E*_*f*_ = 80, 71, and 64 meV, respectively. The corresponding 1D Re(*E*_*z*_) profiles along the propagation direction, highlighting the amplitude decay, are shown in Figure 4b. The resulting propagation lengths are *L*_*p*_ = 10.32, 7.95, and 7.24 μm for *η* = 1/2, 3/5, and 2/3, respectively, indicating that a smaller duty cycle requires a higher *E*_*f*_ to strengthen coupling between adjacent nanoribbons. However, increasing *E*_*f*_ also weakens mode confinement (i.e., increases *λ*_*p*_). To assess other geometric dimension, we further examine the effect of metasurface thickness. For *η* = 3/5 and *ω* = 925 cm^−1^, increasing the thickness from *h* = 100–110 nm requires a slight increase in *E*_*f*_ from 76 to 77 meV to recover the optimal coupling condition, yielding *L*_*p*_ = 7.28 μm. This behavior can be attributed to the *E*_*f*_‐dependent evanescent‐field extent of graphene plasmons, where a higher *E*_*f*_ increases the transverse decay length and therefore enhances coupling with MgTeMoO_6_ phonon polaritons. In contrast, a larger *h* generally reduces *L*_*p*_ due to stronger scattering losses in a taller metasurface. As a result, coherent coupling can be maintained by tuning *E*_*f*_ as the geometric dimensions vary, demonstrating the robustness of the proposed structure to obtain polariton canalizations.

Next, we assess the impact of carrier mobility. For *μ* = 0.1 m^2^/V·s, the Re(*E*_*z*_) distributions at *E*_*f*_ = 76 and 93 meV are shown in Figure 5a,b, respectively. The coupling degradation at *μ* = 0.1 m^2^/V·s can be recovered to the coherent regime (*μ* = 0.5 m^2^/V·s, *E*_*f*_ = 76 meV) by increasing *E*_*f*_ to 93 meV. Similarly, for *μ* = 1.0 m^2^/V·s, coherent coupling is achieved at *E*_*f*_ = 80 meV (Figure 5c,d for *E*_*f*_ = 76 and 80 meV, respectively). Notably, the deviation from the coherent regime at *μ* = 1.0 m^2^/V·s is smaller than that at *μ* = 0.1 m^2^/V·s (compare Figure 5a,c), and therefore requires a smaller *E*_*f*_ adjustment.

**FIGURE 5:**
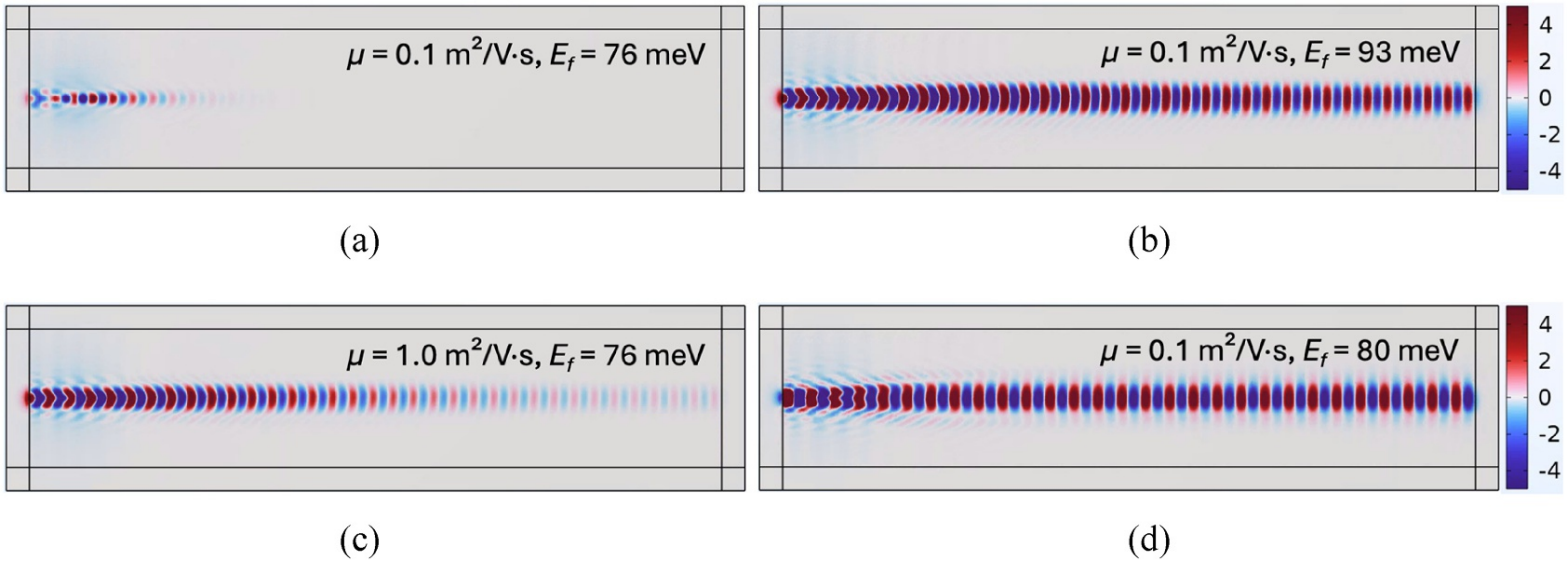
Carrier‐mobility dependence of canalized polaritons. Re(*Ez*) field distributions in the *x*–*y* plane at *ω* = 924 cm^−1^ for different graphene carrier mobilities (*μ*) and Fermi levels (*Ef*): (a) *μ* = 0.1 m^2^/V·s, *Ef* = 76 meV; (b) *μ* = 0.1 m^2^/V·s, *Ef* = 93 meV; (c) *μ* = 1.0 m^2^/V·s, *Ef* = 76 meV; and (d) *μ* = 1.0 m^2^/V·s, *Ef* = 80 meV. Results are obtained with *p* = 75 nm, *w* = 45 nm, *g* = 30 nm, and *h* = 100 nm.

Assessing experimental feasibility, we simulate antenna‐launched excitation of canalized polaritons (Figure 6a). A gold rod antenna serves as a near‐field emitter and is driven by *p*‐polarized plane waves aligned with the antenna's long axis. The antenna concentrates highly confined SPPs at its 300‐nm‐diameter tip, which then couple to the canalized HPPhP mode. Figure 6b shows the simulated Re(*E*_*z*_) distributions at *ω* = 923, 924, and 925 cm^−1^ with *E*_*f*_ = 82, 76, and, 71 meV, respectively, at the parameters *p* = 75 nm, *η* = 3/5, and *h* = 100 nm; the antenna outline is indicated by a yellow boundary. The fields exhibit canalized propagation parallel to the antenna axis, consistent with excitation by an ideal dipole source. The extracted beam widths are 550, 540, and 515 nm for *ω* = 923, 924, and 925 cm^−1^, respectively. These values are consistent with those obtained under dipole excitation, although they are moderately larger. Additionally, the transverse profiles of Re(*E*_*z*_) show that the canalized beams excited by the antenna maintain excellent collimation. These results support the experimental feasibility of the proposed design for realizing long‐range canalized polaritons across a broad range of vdW platforms.

**FIGURE 6:**
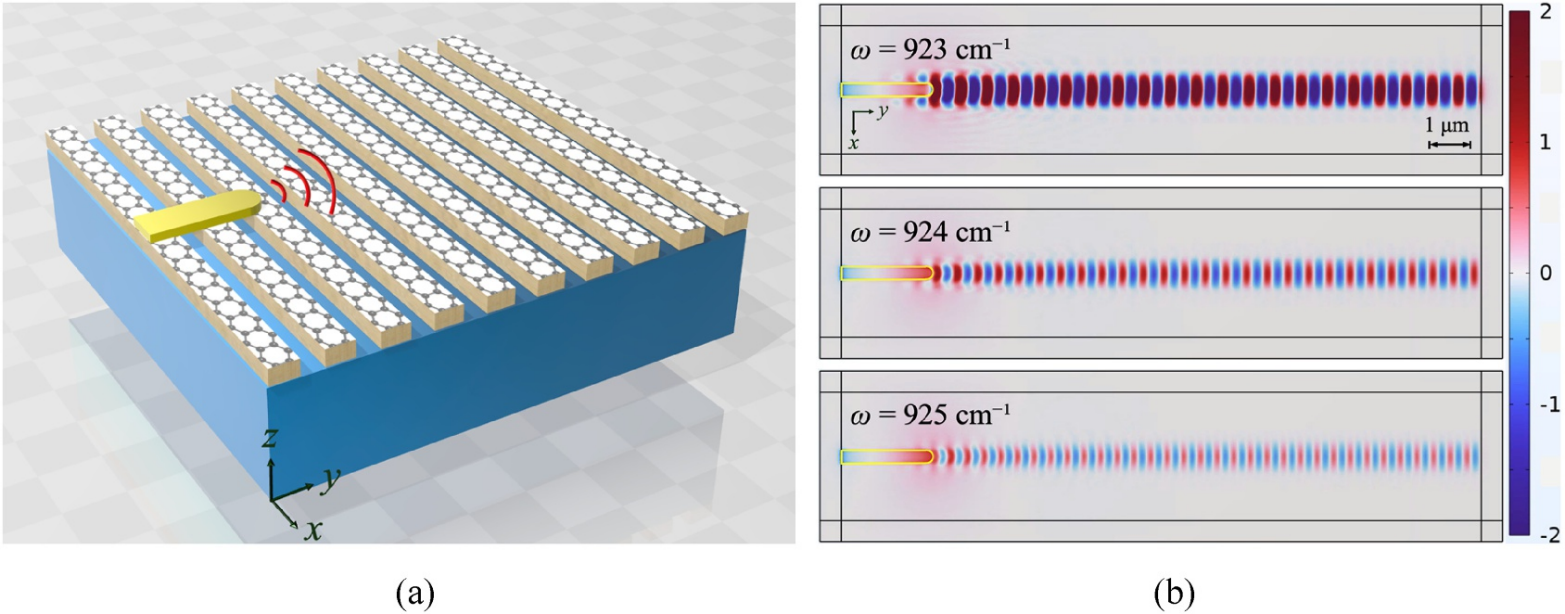
Simulated antenna‐launched excitation of canalized polaritons. (a) Schematic of the antenna‐launch configuration used to excite canalized polaritons; the gold rod antenna is shown in gold color. (b) Simulated Re(*E*_*z*_) field distributions at *ω* = 923 (*E*_*f*_ = 82 meV), 924 (*E*_*f*_ = 76 meV), and 925 cm^−1^ (*E*_*f*_ = 71 meV) for *p* = 75 nm, *η* = 3/5, and *h* = 100 nm. The yellow outline indicates the antenna boundary.

Finally, we examined the fabrication tolerance and the sensitivity to the Fermi level, as shown in Figure 7. The reference propagation length is *L*_*p*_ = 7.95 μm, obtained at *p* = 75 nm, *w* = 45 nm, *g* = 30 nm, *h* = 100 nm, *E*_*f*_ = 71 meV, and *ω* = 925 cm^−1^. We observe that *L*_*p*_ remains above 4 μm over the range ∣Δ*E*_*f*_∣ < 5 meV, indicating that the polariton mode is relatively robust against moderate fluctuations in *E*_*f*_. In contrast, *L*_*p*_ is more sensitive to variations in the gap width. When the gap becomes narrower (Δ*g* < 0), the change in *L*_*p*_ is moderate, remaining within about 2 μm of the reference value. However, when the gap becomes wider (Δ*g* < 0), *L*_*p*_ decreases rapidly and drops to nearly 1 μm at Δ*g* = + 5 nm. The results show that *L*_*p*_ is more tolerant to Fermi‐level fluctuations than to positive deviations in the gap width.

**FIGURE 7:**
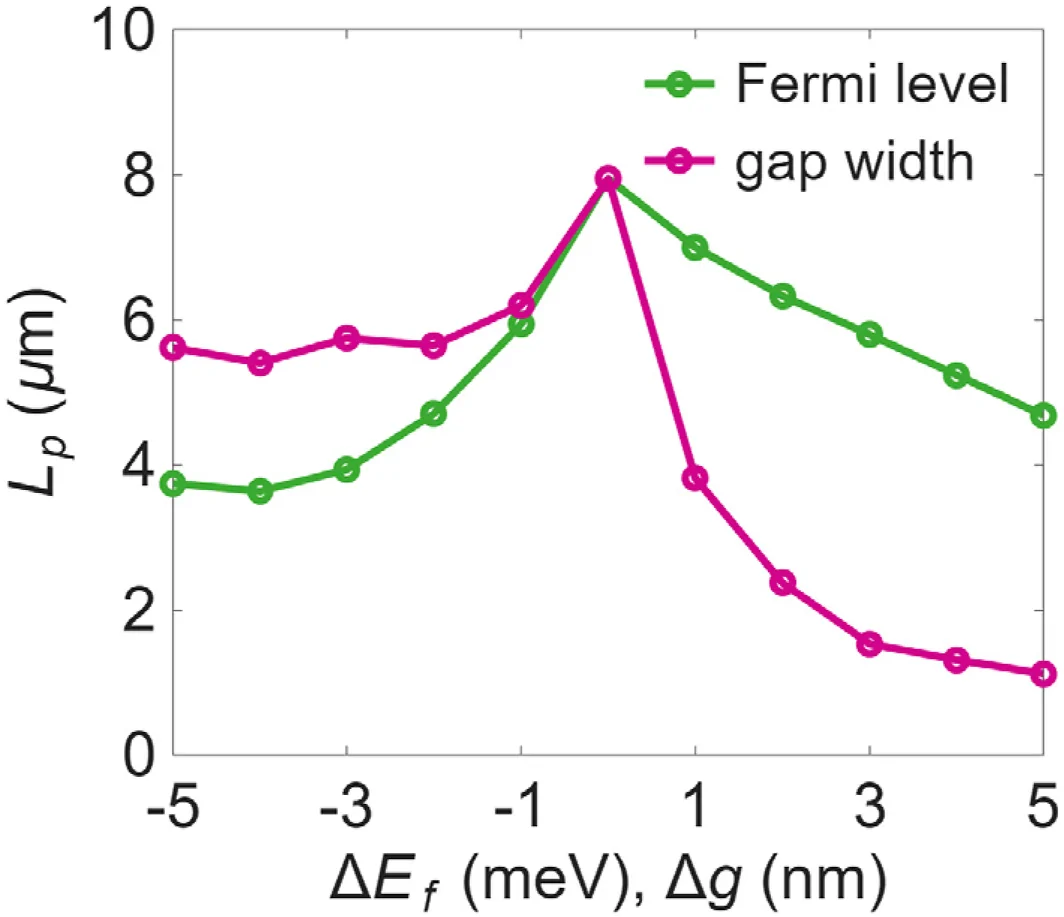
Dependence of *L*_*p*_ on fabrication tolerance and Fermi‐level variation. *L*_*p*_ is plotted as a function of the deviations in gap width Δ*g* and Fermi level Δ*E*_*f*_, illustrating the sensitivity of *L*_*p*_ of the canalized polariton at the parameters *p* = 75 nm, *w* = 45 nm, *g* = 30 nm (Δ*g* = 0), and *h* = 100 nm, *E*_*f*_ = 71 meV (Δ*E*_*f*_ = 0), and *ω* = 925 cm^−1^.

## 5 Conclusions

We propose an MgTeMoO_6_ metasurface covered with graphene nanoribbons on the ridge tops to realize long‐range canalized polaritons with a propagation length of *L*_*p*_ = 7.95 μm and a correspondingly long lifetime of *τ* = 86 ps at the operating frequency *ω* = 925 cm^−1^. This substantial improvement markedly enhances light–matter interactions and outperforms previously reported vdW canalization platforms. The ≈ 20‐fold increase in *L*_*p*_ relative to the bare MgTeMoO_6_ metasurface enables practical opportunities for nanophotonic integrated circuits, super‐resolution imaging, and mid‐IR chemical sensing. The observed enhancement arises from hybridization between the surface plasmon polaritons of graphene nanoribbons and the phonon polaritons of the MgTeMoO_6_ metasurface. By tuning the graphene Fermi level, coherent coupling is achieved, suppressing radiative leakage and allowing the propagation to be primarily limited by material absorption. Using the optimal Fermi levels, we systematically investigate the propagation characteristics across the canalization band *ω* = 922–927 cm^−1^ and further examine the spectral, geometric, and carrier mobility dependences of the canalized modes. To assess experimental feasibility, we numerically simulate an antenna‐launched excitation scheme. The resulting field patterns and propagation characteristics closely match those obtained with an ideal dipole source, supporting the practicality of the proposed design. Overall, our results provide a general strategy for achieving long‐range canalized polaritons and can be extended to other vdW polaritonic platforms, offering a promising route toward advanced mid‐IR nanophotonics and imaging technologies.

## Author Contributions

This work was finished solely by Chia‐Chien Huang.

## Conflicts of Interest

The author declares no conflicts of interest.

## Data Availability

The data that supports the findings of this study are available in the supplementary material of this article.
